# Congenital lobar emphysema

**DOI:** 10.1590/0100-3984.2016.0224

**Published:** 2018

**Authors:** Felipe Mussi von Ranke, Heloisa Maria Pereira Freitas, Vanessa Dinoá, Fernanda Miraldi, Edson Marchiori

**Affiliations:** 1 Universidade Federal do Rio de Janeiro (UFRJ), Rio de Janeiro, RJ, Brazil.

*Dear Editor,*

**Figure 1:**
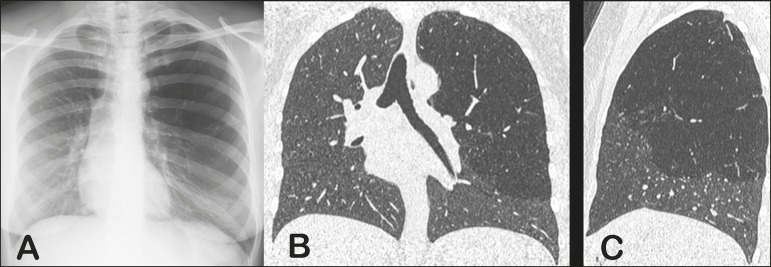
Anteroposterior chest X-ray (**A**) showing radiolucency and hyperinflation of the upper two thirds of the left lung. HRCT with coronal and sagittal reconstructions (**B** and **C**, respectively) showing hyperinflation of the left upper lobe of the lung, as well as vessel attenuation.

A 34-year-old asymptomatic woman underwent a chest radiography examination as an admission requirement for a new job. The X-ray showed focal hyperlucency in the left upper lobe of the lung (Figure 1A). High-resolution computed tomography (HRCT) was performed to confirm the findings (Figures 1B and 1C). The HRCT findings were characteristic of congenital lobar emphysema (CLE).

The evaluation by imaging methods in pediatrics has been the subject of a series of recent publications in the radiology literature of Brazil^(^^1^^-^^6^^)^. CLE is characterized by hyperinflation of one or more lung lobes in the absence of extrinsic bronchial obstruction^(^^7^^)^. It is a rare disease and its incidence is 20-30 cases/1000 births, most commonly affecting a single lobe of the lung (typically the left upper lobe), although multiple lobes or specific lobar segments may be involved^(^^7^^,^^8^^)^. The disease has a variety of causes, including bronchial cartilage deficiency (bronchomalacia) and endobronchial lesions, resulting in narrowing of the airway lumen and obstruction with air trapping, as well as progressive lobar overexpansion, usually with compression of the remaining areas of the ipsilateral lung^(^^9^^)^.

CLE is generally diagnosed during early infancy, presenting with persistent progressive respiratory distress. It is known that CLE can occur in association with other malformations, especially cardiac malformations, which are present in 20% of cases^(^^7^^)^. In rare cases, it is diagnosed in adulthood and must be differentiated from other causes of localized pulmonary hyperlucency, because the treatments differ^(^^9^^)^. In such cases, the patients are usually asymptomatic and the disease can go unnoticed, resulting in underestimation of the true incidence of this condition.

Conventional chest X-rays are typically used in order to establish the diagnosis of CLE, showing a unilateral hyperlucent hemithorax. This finding is also present in a variety of other conditions, which include tension pneumothorax-the main differential diagnosis on routine chest radiography^(^^7^^)^-as well as bullous disease, pneumatocele, Swyer-James syndrome, endobronchial mass, unilateral pulmonary agenesis, proximal interruption of the pulmonary artery, scimitar syndrome, diaphragmatic hernia, and Poland syndrome^(^^8^^)^. It can also exclude an intrathoracic mass or vascular ring. HRCT is useful for confirming radiographic findings, delineating the affected lobe and showing relative narrowing of the bronchus associated with hyperinflation and attenuated vessels in the hyperlucent lobe, which facilitate the differential diagnosis.

Lobectomy is the treatment for nearly all cases of CLE with respiratory distress. According to Karnak et al.^(^^10^^)^, lobectomy is the recommended treatment for CLE in all infants under two months of age and in older infants who present with severe respiratory symptoms. Apparently, the earlier the presentation is, the greater is the need for surgery. Conservative management, with close outpatient follow-up, can be used in older children who present with mild to moderate symptoms. Because our patient had remained asymptomatic throughout her life, her case was managed with clinical and radiographic follow-up.
